# Efficient Visible-Light Photocatalysis of TiO_2-δ_ Nanobelts Utilizing Self-Induced Defects and Carbon Doping

**DOI:** 10.3390/nano11061377

**Published:** 2021-05-23

**Authors:** Dong-Bum Seo, Sung-Su Bae, Eui-Tae Kim

**Affiliations:** Department of Materials Science & Engineering, Chungnam National University, Daejeon 34134, Korea; sdb987@naver.com (D.-B.S.); bss1007@naver.com (S.-S.B.)

**Keywords:** TiO_2_, photocatalysis, nanobelts, chemical vapor deposition

## Abstract

Efficient visible-light photocatalysis was realized by exploring self-induced defect states, including the abundant surface states of TiO_2-δ_ nanobelts synthesized through metal–organic chemical vapor deposition (MOCVD). The TiO_2-δ_ nanobelts exhibited two strong defect-induced absorption peaks at 2.91 and 1.92 eV, overlapping with the conduction band states so that photoexcited carriers can contribute effectively for the photocatalysis reaction. To further enhance visible-light photocatalytic activity, carbon atoms, the by-product of the MOCVD reaction, were self-doped at the judiciously determined growth conditions. The resulting visible-light photocatalysis suggests that the large surface area and consequent high concentration of the surface states of the TiO_2-δ_ nanobelts can be effectively utilized in a wide range of photocatalysis applications.

## 1. Introduction

Efficient photocatalytic activity of TiO_2_ has attracted extensive interest for various photochemistry applications, such as in the elimination of pollutants [1,2,3] and the splitting of water for hydrogen fuel [4,5], as well as for their applications in solar-cell electrodes [6,7]. Recently, much attention has been focused on the use of low-dimensional TiO_2_ nanostructures, such as nanotubes [7], nanowires [8], and nanobelts [9,10], with high specific surface areas to further enhance their reactivity. Particularly, TiO_2_ nanobelts have been extensively studied in various applications such as photocatalysis, solar cells, gas sensors, and biosensors due to their unique morphology, fast charge transport in the axial direction, short travel distance from inside to surface, and strong surface reactivity [9,10]. However, the wide band gap energy (~3.0–3.2 eV) of TiO_2_ limits its excellent photocatalytic reactivity to UV light. For practical applications, special attention has to be paid to the development of 2D TiO_2_ nanostructures that can yield high reactivity under visible light. Visible-light photocatalysis possesses a tremendous advantage given that the main part (~43%) of the solar spectrum and even room lighting can be utilized. Toward this end, considerable efforts have been exerted to extend the reactivity of TiO_2_ to a visible-light regime via the doping of a number of cationic metals [2,11] or anionic non-metals such as nitrogen [3] and carbon [5,12]. Among them, carbon doping is considered one of the most effective dopants for visible-light photocatalytic reaction [5,12,13,14,15,16]. However, simple self-doped approaches have been limited to thin films [13,14], nanoparticles [15], and nanotubes [16]. Little information is available on realizing visible-light photocatalysis in 2D TiO_2_ nanostructures. Moreover, meaningful visible-light photocatalysis has not so far been reported by utilizing surface defect states, which are abundant especially for 2D TiO_2_ nanostructures with high specific surface areas. It is known that coordinatively unsaturated Ti sites, such as Ti^3+^, on the surface are photocatalytically active [17]. As such, they can be hydroxylated in organic medium or ambient at room temperature [18,19].

In this study, we explored self-induced defect states, such as the abundant surface states of TiO_2-δ_ nanobelts synthesized by metal–organic chemical vapor deposition (MOCVD), for visible-light photocatalysis. In addition, we utilized carbon, which was the by-product of the MOCVD reaction, as a dopant to further enhance visible-light photocatalysis. The synthesis of TiO_2-δ_ nanobelts was carried out without using an oxidant, and the synthesis temperature was judiciously determined at 510 °C, which was slightly lower than the complete decomposition temperature (>527 °C) [20] of the Ti precursor. In this manner, the significant amount of carbon could reside in the grown TiO_2-δ_. This approach of utilizing self-induced defects and carbon doping is very simple and effective to realize efficient visible-light photocatalysis of TiO_2_ nanostructures.

## 2. Materials and Methods

TiO_2-δ_ nanobelts were grown on bare Si(001) and glass substrates for 10 min by MOCVD. The growth process was carried out without the use of any metal catalysts at a high density. The (C_11_H_19_O_2_)_2_(C_3_H_7_O)_2_Ti (Kosundo) was used as a Ti precursor and bubbled at 210 °C with an Ar gas flow of 150 SCCM (SCCM denotes standard cubic centimeters per minute). The growth temperature, pressure, and total Ar flow were fixed at 510 °C, 10 Torr, and 300 SCCM, respectively. For the annealed TiO_2_ nanobelt sample, as-grown TiO_2-δ_ nanobelts were rapid-thermal annealed at 600 °C under an O_2_ gas flow of 200 SCCM for 10 min. After loading as-grown TiO_2-δ_ nanobelt samples in the RTA chamber, the sample temperature was increased at the rate of 100 °C/min to 600 °C. In addition, as a counterpart of the nanobelt samples, the TiO_2_ thin film was grown under the exactly same conditions as the TiO_2-δ_ nanobelt sample, except for an O_2_ gas supply of 50 SCCM.

The samples were characterized by using scanning electron microscopy (SEM, JEOL JSM-7000F, Zhaodao City, Japan), an X-ray diffractometer (XRD, Rigaku D/MAX-RC, Cu *K_α_* radiation, and a Ni filter, Tokyo, Japan), transmission electron microscopy (TEM, JEOL 2100F), X-ray photoelectron spectroscopy (XPS, ESCALAB 200R, Waltham, MA, USA), and UV–VIS spectroscopy (Shimadzu UV-2450, Tokyo, Japan). Photocatalytic activity was studied by measuring the decomposition of methylene blue (MB) in an aqueous solution using UV–VIS spectroscopy. The initial concentration of MB solution was 10^−5^ mol∙L^−1^. The TiO_2_ samples were dipped in the MB solution in a Petri dish and illuminated with visible and UV lights. The white light (100 mW∙cm^−2^) of a common room lighting bulb and the UV light of 365 nm (10 mW∙cm^−2^) were used as visible light and UV irradiation sources, respectively.

## 3. Results

**Figure 1:**
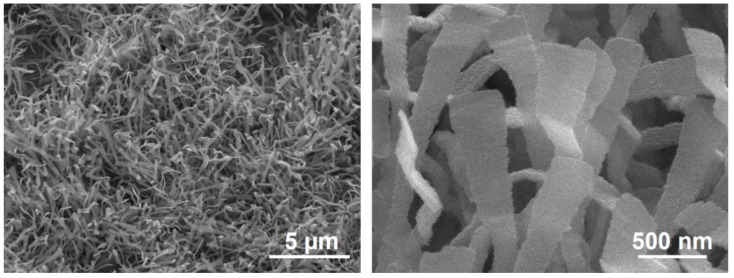
SEM images of the TiO_2-δ_ nanobelts grown on a bare Si (001) wafer.

Figure 1 shows typical SEM images of TiO_2-δ_ nanobelts grown on a bare Si (001) wafer for 10 min. The nanobelts appear as thin rectangular belts at a high density. The nanobelts have a length of ~5–8 µm, while the width and thickness are ~300–500 nm and ~20–50 nm, respectively. We have previously demonstrated the synthesis of such TiO_2-δ_ nanobelts and nanosheets governed by a vapor–solid (V–S) growth mechanism [21,22]. It should be emphasized that the various TiO_2-δ_ nanobelts and nanosheets, which we synthesized via the approach presented here, demonstrated efficient visible-light photocatalysis characteristics.

In this work, we focused on the fundamental issue to reveal the origin of the observed visible-light photocatalysis of the TiO_2-δ_ nanobelts as shown in Figure 1 (referred to here as TiO_2-δ_ nanobelts). To gain insight on such an origin, the TiO_2-δ_ nanobelts were rapid-thermal annealed at 600 °C under an O_2_ environment for 10 min (hereby referred to as annealed TiO_2_ nanobelts). The annealed TiO_2_ nanobelt sample was an insulator with an electrical resistivity value that was ~1000 times larger than that of the TiO_2-δ_ nanobelt sample due to the significant decrease in oxygen vacancies. Note that the TiO_2-δ_ nanobelts were grown without using an oxidant. As a result, the grown TiO_2-δ_ was significantly reduced, with a very low resistivity of a few Ω cm. Likewise, an insulating TiO_2_ thin film with a thickness of ~0.1 µm was studied as a counterpart of the nanobelt samples.

**Figure 2:**
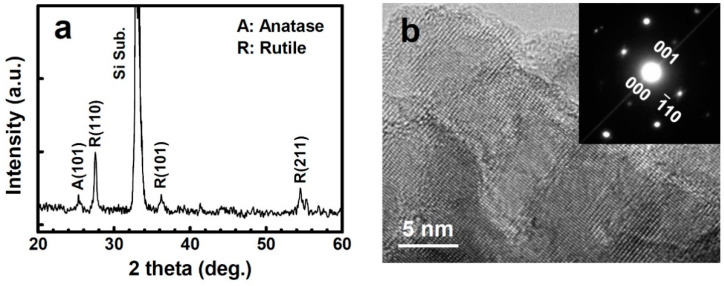
(**a**) The XRD pattern of the TiO_2-δ_ nanobelt sample. (**b**) A typical high-resolution TEM lattice image of the TiO_2-δ_ nanobelts and its SAD pattern (inset).

Figure 2a shows the XRD pattern of the TiO_2-δ_ nanobelt sample. The rutile phases are predominantly observed along with a small anatase (101) peak. We note that the annealed TiO_2_ nanobelt sample did not show any discernable change in the XRD pattern and SEM morphology. The lattice image of the high-resolution TEM reveals that the nanobelts consist of ~10–30 nm size nanocrystallites, which are well aligned with one another (Figure 2b). As seen in the inset of Figure 2b, the selected area diffraction (SAD) pattern confirms that the nanocrystallites are oriented in a specific crystallographic direction. One of the most dominantly observed SAD patterns is the [110] zone-axis rutile phase indicating that (110) planes are the surface exposed plane. Note that the XRD pattern shows the rutile (110) planes to be the most exposed plane as well. It is known that the TiO_2_ (110) plane has defect stoichiometry yielding Ti^3+^ defect sites, which are photocatalytically active [17].

**Figure 3:**
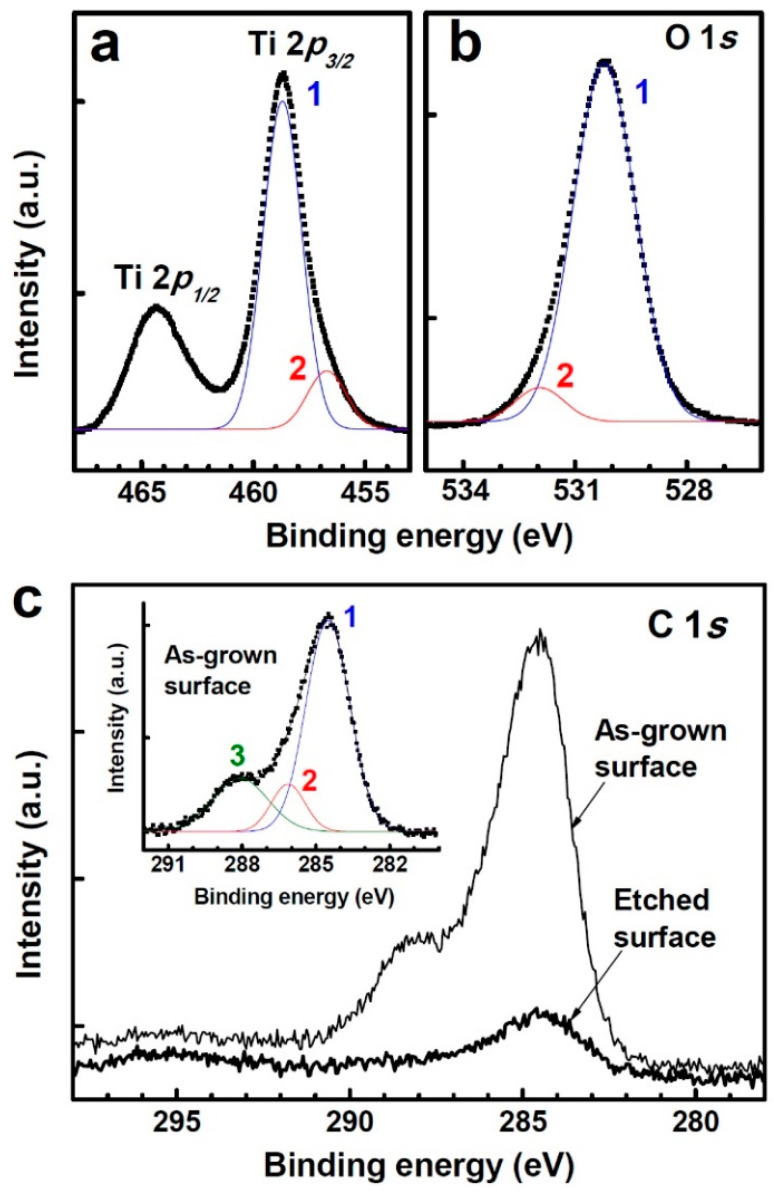
(**a**) The XPS spectrum of the Ti 2*p* core level of the TiO_2-δ_ nanobelts. (**b**) The O 1*s* spectrum resolved into two peaks at 530.2 (peak 1) and 531.9 eV (peak 2). (**c**) The C 1*s* spectrum of the as-grown and the etched surface (~10 Å). The inset shows three deconvoluted peaks of the C 1*s* spectrum of the as-grown surface: Peak 1 at 284.5 eV, peak 2 at 286.1 eV, and peak 3 at 288.0 eV correspond to non-functionalized C–C bond, the elemental carbon atoms bonded to Ti and to a single oxygen atom, and carbonate species, respectively.

**Figure 4:**
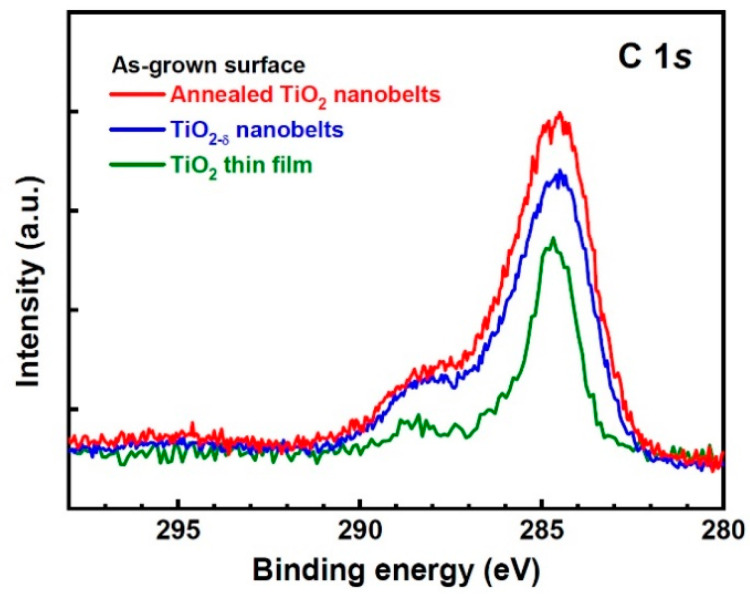
The XPS C 1*s* spectra of the TiO_2-δ_ nanobelts, the annealed TiO_2_ nanobelts, and the TiO_2_ thin film (as-grown surface).

The chemical states and carbon doping of the TiO_2-δ_ nanobelts, the annealed TiO_2_ nanobelts, and the TiO_2_ thin film were studied using XPS. As seen in Figure 3a, the Ti 2*p_3/2_* peak of the TiO_2-δ_ nanobelts has a small shoulder at a lower binding energy side and can be deconvoluted into two Gaussian components peaked at 458.7 and 456.7 eV (referred to as peak one and two, respectively). Peak one and two can be assigned to Ti^4+^ (normal state) and Ti^3+^, respectively. The peak separation (2.0 eV) of peak one and two is in agreement with the reported value (~1.8–1.9 eV) of Ti^4+^ and Ti^3+^ states [19,23]. The area ratio of peak two to one is ~0.18, indicating that a significant amount of Ti^3+^ states exists. The ratio of peak two to one was not noticeably changed in the annealed TiO_2_ nanobelts. As seen in Figure 3b, the O 1*s* peak of the TiO_2-δ_ nanobelts can be resolved into two peaks as well. Peak one (530.2 eV) and peak two (531.9 eV) correspond to the oxygen bonded to Ti (Ti–O) and the oxygen from hydroxyl species (OH^−^), respectively [19,23]. The hydroxyl species can result from the existence of Ti^3+^ states, which are photocatalytically active and can be hydroxylated in organic medium or ambient at room temperature [17,18,19]. Figure 3c shows that carbon (C) significantly resides on the as-grown surface of the TiO_2-δ_ nanobelts. As seen in the inset of Figure 3c, peak one at 284.5 eV corresponds to non-functionalized C–C bond [24]. Peak two at 286.1 eV can be attributed to the elemental carbon atoms bonded to Ti (substitutional carbon doping) and to carbon atoms linked to a single oxygen atom (C–OH) [12,24]. Peak three at 288.0 eV corresponds to carbonate species [12,24]. After surface etching (~10 Å) by Ar^+^ ion sputtering, the overall peak intensity is remarkably decreased, and peak three which arose from carbonate species is diminished. However, it is clearly seen that peak one and two still exist significantly inside the TiO_2-δ_ nanobelts. As seen in Figure 4, the annealed TiO_2_ nanobelts exhibited a C 1*s* spectrum similar to the TiO_2-δ_ nanobelts, suggesting that a significant amount of carbon still remained in the TiO_2_ nanobelts after annealing. In addition, the Ti 2*p_3/2_* and O 1*s* peaks of the annealed TiO_2_ nanobelts were almost the same as those of the TiO_2-δ_ nanobelts (Figure S1 in Supporting Information). Compared with the nanobelt samples, the TiO_2_ thin film showed a very weak shoulder peak at 288.0 eV (Figure 4). After surface etching (~10 Å), the carbon peak of the TiO_2_ thin film almost disappeared (Figure S2).

**Figure 5:**
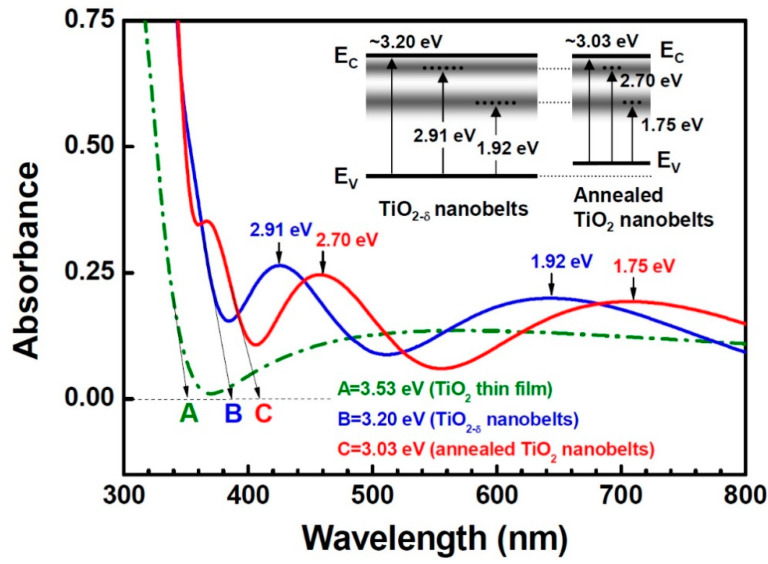
The UV–VIS spectra of the TiO_2-δ_ nanobelts, the annealed TiO_2_ nanobelts, and the TiO_2_ thin film. The inset shows the schematics of energy levels and associated optical absorption transitions of the TiO_2-δ_ and the annealed TiO_2_ nanobelts.

Figure 5 shows the optical absorption spectra of the TiO_2-δ_ nanobelts, the annealed TiO_2_ nanobelts, and the TiO_2_ thin film. The TiO_2-δ_ nanobelts show an optical absorption threshold at 3.20 eV (point B), whereas the annealed TiO_2_ nanobelts show another absorption threshold at 3.03 eV (point C) along with that at ~3.20 eV. The second absorption threshold (point C) is the result of an enhanced substitutional carbon doping by annealing. In relation, Khan et al. [5] reported that rutile thin film showed two absorption thresholds due to carbon doping by natural gas flame pyrolysis. We also note that the TiO_2-δ_ nanobelts show two strong broad absorption peaks centered at 2.91 and 1.92 eV. There is a greater likelihood that these absorption peaks can be attributed to bands of surface states and interstitial Ti^3+^ ions [25,26]. Other workers reported that surface states were shallow intragap states placed at ~0–1 eV below the conduction band edge [25,26]. The possibility of carbon contribution cannot be completely ruled out, but calculations [3] show that the energy states by carbon substitutional doping are too deep in the gap to overlap with the conduction band states of TiO_2_. Unlike reduced anatase TiO_2-δ_ [27], these absorption peaks cannot be due to oxygen vacancies either because the annealed TiO_2_ nanobelts also show similar absorption peaks at 2.70 and 1.75 eV. These peaks of the annealed TiO_2_ nanobelts are red-shifted by 0.21 and 0.17 eV, respectively, with respect to those of the TiO_2-δ_ nanobelts. The red-shifted energies correspond to the absorption-threshold shift from point B to point C (0.17 eV) after annealing. This behavior can be explained by a shift of the valence band edge as seen in the inset of Figure 5. It was reported that the narrowed bandgap energy (~0.14 eV) of carbon-doped TiO_2_ powders was attributed to the shifting of the valence band edge [12]. It should be emphasized that the defect-state bands, which peaked at 2.91 eV of the TiO_2-δ_ nanobelts and 2.70 eV of the annealed TiO_2_ nanobelts, satisfy the important requirements of visible-light photocatalysis. The energy states in the gap can create photoexcited carriers by visible-light absorption. Moreover, the defect-state bands overlap sufficiently with the conduction band states of TiO_2_ so that photoexcited carriers can transfer to reactive sites at the surface for photocatalysis. The high specific surface area and very thin thickness (~20–50 nm) of the nanobelts can be a great advantage for efficient carrier transfer to surface without recombination. Meanwhile, the TiO_2_ thin film shows a larger absorption threshold energy of 3.53 eV (point A), which is most probably related to the onset of direct optical transitions. The larger energy may be a result of anatase structure and less carbon content. We note that although the TiO_2_ thin film has very broad absorption over the visible-light regime, the broad band did not overlap with the band states of TiO_2_.

**Figure 6:**
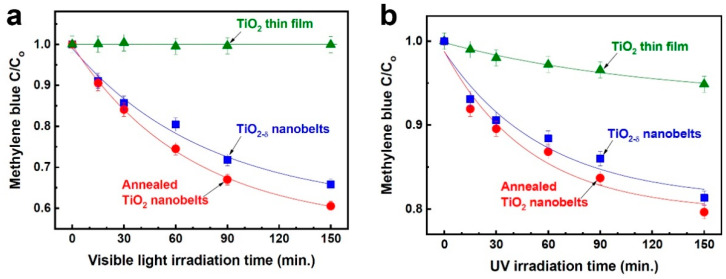
Photocatalytic properties of the TiO_2-δ_ nanobelts, the annealed TiO_2_ nanobelts, and the TiO_2_ thin film. The ratio of the remaining concentration to the initial concentration of methylene blue in an aqueous solution as a function of visible-light irradiation time (**a**) and UV irradiation time (**b**).

Photocatalytic activity was evaluated by measuring the decomposition of methylene blue in an aqueous solution. As seen in Figure 6a, the TiO_2-δ_ nanobelts decompose methylene blue effectively through photocatalytic reaction under visible-light irradiation, whereas the TiO_2_ thin film does not show any photocatalytic activity. Visible-light photocatalysis is further enhanced for the annealed TiO_2_ nanobelt sample, indicating that the observed visible-light photocatalysis cannot be due to oxygen vacancies. Under UV irradiation, the TiO_2-δ_ nanobelts also show much stronger photocatalytic activity than the TiO_2_ thin film (Figure 6b) because of its much larger specific surface area. Moreover, the annealed TiO_2_ nanobelts show a little bit better photocatalytic activity than the TiO_2-δ_ nanobelts because of the improved structural quality via annealing.

## 4. Conclusions

In conclusion, we demonstrated the efficient photocatalysis of TiO_2-δ_ nanobelts synthesized by MOCVD in both visible and UV light radiation. Visible-light photocatalysis was realized by utilizing self-induced defect states, including the abundant surface states of nanobelts and self-doped carbon atoms. The carbon atoms, the by-product of MOCVD, could be self-doped in the TiO_2-δ_ nanobelts by determining the growth conditions judiciously. The approach we used was simple and very efficient for visible-light photocatalysis, and the TiO_2-δ_ nanobelts can be effectively utilized in a wide range of photocatalysis applications, such as the elimination of pollutants and the electrodes of solar cells.

## Data Availability

Data are available in the main text.

## Supplementary Materials

The following are available online at https://www.mdpi.com/article/10.3390/nano11061377/s1, Figure S1: XPS Ti 2*p* and O 1*s* spectra of the annealed TiO_2_ nanobelts title, Figure S2: XPS C 1*s* spectra of the TiO_2-δ_ nanobelts, the annealed TiO_2_ nanobelts, and the TiO_2_ thin film after surface etching (~10 Å).
