# Supplementary material for: Efficient Visible-Light Photocatalysis of TiO2-δ Nanobelts Utilizing Self-Induced Defects and Carbon Doping
Source: Nanomaterials (Basel). 2021 May 23;11(6):1377. doi: 10.3390/nano11061377 (PMC8224642; doi:10.3390/nano11061377)
Supplement: Supplementary file 1 [file nanomaterials-11-01377-s001.zip › nanomaterials-1191749-SI.pdf]

Supporting Information

Efficient Visible-Light Photocatalysis of $TiO_{2-\delta}$ Nanobelts Utilizing Self-Induced Defects and Carbon Doping

Dong-Bum Seo, Sung-Su Bae and Eui-Tae Kim *

Department of Materials Science & Engineering, Chungnam National University, Daejeon 34134, Korea; sdb987@naver.com (D.-B.S.); bss1007@naver.com (S.-S.B.)

* Correspondence: etkim@cnu.ac.kr

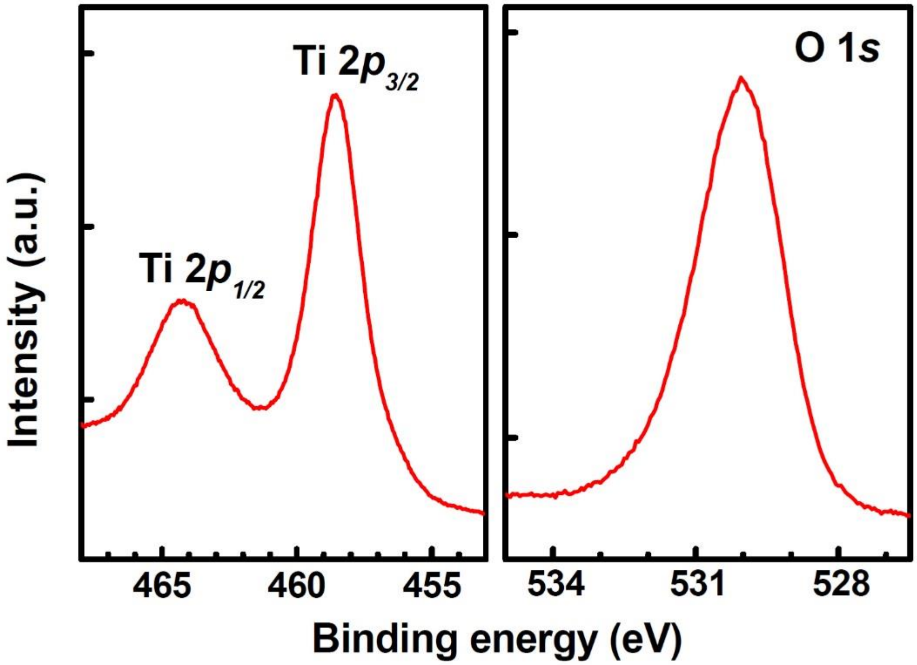

Figure S1. XPS Ti 2*p* and O 1*s* spectra of the annealed TiO_2 nanobelts.

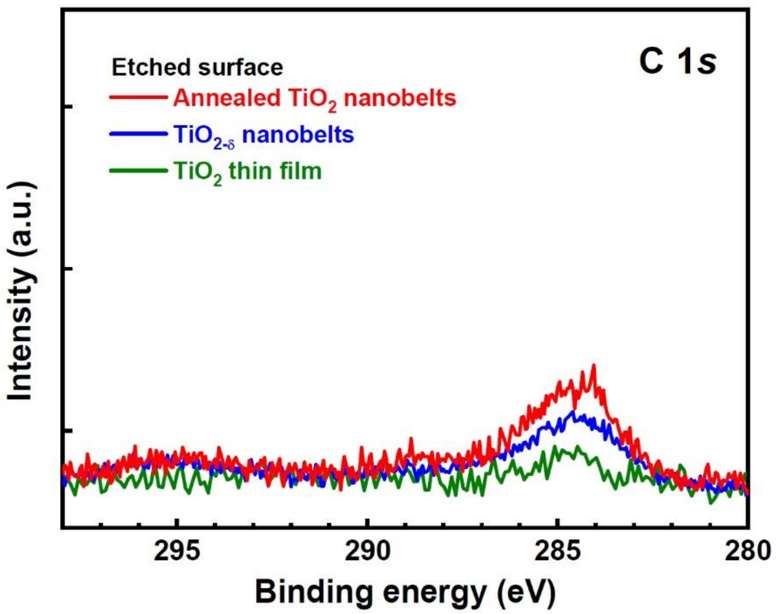

Figure S2. XPS C 1*s* spectra of the TiO_{2-} nanobelts, the annealed TiO_2 nanobelts, and the TiO_2 thin film after surface etching (~10 Å).
